# The Performance Gap in Sport Can Help Determine Which Movements Were Most Essential to Human Evolution

**DOI:** 10.3389/fphys.2019.01412

**Published:** 2019-11-19

**Authors:** Collin Carroll

**Affiliations:** Columbia University, New York, NY, United States

**Keywords:** performance gap, sex-blind, musculoskeletal, adaptations, track and field, sex equity score

## Abstract

Men outperform women in sports that require muscular strength and endurance, but the magnitude of this performance gap (PG) does not appear to be constant; that is, the PG between men and women is greater in some sports than it is in others. Here, we examine the size of this gap within the realm of track and field by comparing the top 50 world-record performances of men to the top 50 records set by women in a number of long-distance running, medium-distance running, short-distance running, and jumping events. While women do not perform at the level of men in any track and field event, the magnitude of the PG trends up or down depending on the type of event. Jumping events exhibit a larger gap between the sexes than do running events, and short-distance running events show a smaller disparity between the sexes than do medium- or long-distance running events. This difference suggests that general sexual dimorphism does not explain why female performance is relatively closer to male performance at some track and field events than others. We hypothesize that this trend can be explained by the presence of sex-blind musculoskeletal adaptations (SBMA’s), which accumulate over generations to reduce the size of the PG in certain movements. We conclude that the selection trend favoring in humans should be explored further to determine whether the PG in sport can indeed be used to determine movements to which the human body is adapted.

## Introduction

Movement is key to an organism’s reproductive success; better quality of movement allows an individual to escape predators and acquire food, heightening its reproductive fitness (Kuhn et al., 2016; Marck et al., 2016). Hominids in particular are capable of a variety of actions, including both arboreal (Venkataraman et al., 2012) and terrestrial movement (Crompton et al., 2010; Roberts et al., 2016), and the notable ability of humans to successfully navigate different environments has led to the remarkable spread of *Homo sapiens* all around the world (Petraglia et al., 2010; Rito et al., 2013). The human body is capable of climbing, short- and long-distance running, swimming, jumping, and many more movements, all of which can be assessed experimentally between individuals as sport.

Sport, specifically track and field, has existed for thousands of years. Recent advancements in exercise science and nutrition have allowed new track and field world records to be consistently achieved throughout the 20th century, but in recent years, the rate of new records has slowed (Matrahazi and Hymans, 2015; Marck et al., 2017). This stagnation of world records suggests that humans are approaching the apex of our species’ ability to perform, which makes current track and field world records a good gauge for the overall capacity of the human body at these established events. And while the rate of new world records has slowed in recent years, a notable performance gap (PG) remains between top-performing men and top-performing women across all track and field events.

Men have a physiological makeup that does indeed advantage them over women in strength- and endurance-based competition (Sandbakk et al., 2018). In addition to a lower body-fat percentage, men have a greater percentage of muscle mass, denser bones, higher testosterone levels, and a longer stride length than women do (Smith and Smith, 2002; Nieves et al., 2004). While women have closed much of the PG throughout the 20th century due to changing cultural norms (Thibault et al., 2010), it appears that the PG has reached an asymptote since the 1980’s (Millard-Stafford et al., 2018). Though men tend to outperform women overall, the magnitude of the PG narrows or widens depending on the sport. Swimming, track and field, and speed skating tend to show a relatively small PG, whereas the gap is considerably larger in weightlifting competition (Thibault et al., 2010). Even within the sport of track and field, the PG demonstrably expands or contracts depending on the event (Matrahazi and Hymans, 2015).

If the general physiological advantages men have over women in sport are the sole contributors to superior male ability in track and field events, we would not expect the PG to be significantly different across different events. That the magnitude of the athletic gap appears to be event-dependent instead suggests that sexual dimorphism is not the only factor contributing to the PG. Absent confounding factors, a smaller athletic gap in a particular event indicates either that female physiology is relatively better-suited to the event, or that there exist sex-blind musculoskeletal adaptations (SBMA’s) shared by men and women which partially negate the general physiological advantage men have over women.

The purpose of this investigation was to determine how the gap that exists between the top 50 records set by males and the top 50 records set by females expands or contracts across different track and field events. These results will be further analyzed through an evolutionary lens. The goal of this analysis will be to determine whether the magnitude of the PG corresponds to established movements considered essential to human evolution, which in turn could determine whether the PG in sport can be used as evidence that SBMA’s exist in humans.

## Materials and Methods

### Sex Equity Score by Event

The sex equity score (SES) data set was collected online from the website of the International Association of Athletics Federations (IAAF, 2018) (Supplementary Data Sheet 1). The top 50 world-record overall performances (independent of athlete) and the top 50 world-record performances by athlete as of May 1, 2018 were taken for both men and for women.

The SES, which is the ratio of the mean of the top men’s world records (MTS_m_) and the mean of the top women’s records (MTS_w_) in the same track and field event, is calculated differently for chronometric and non-chronometric events, as follows. The initial result is multiplied by 100 to better illustrate inter-event differences.

forchronometric(running)events,MTS<mMTSwSES=(MTS/mMTS)w*100fornon-chronometric(jumping)events,MTS>mMTSwSES=(MTS/wMTS)m*100

The SES was calculated 12 times for each event. The first six calculations compared the MTS_m_ to the MTS_w_ for world-record performances independent of athlete. The MTS_m_ for all 50 male performances was compared to the MTS_w_ for all 50 female performances for the first SES calculation. Then, the top 50 performances for both men and women were broken into five quintiles: performances 1–10, 11–20, 21–30, 31–40, and 41–50. The SES was calculated laterally for each group, so that the MTS_m_ of the top 10 male performances (the first male quintile) was compared to the MTS_w_ of the top 10 female performances (the first female quintile). Next, the MTS_m_ of male performances 11–20 was compared to the MTS_w_ of female performances 11–20; this calculation was repeated for all five quintiles. This procedure was repeated for the next six SES calculations, except these calculations compared the MTS_m_ to the MTS_w_ for world-record performances by athlete so that no athlete is represented more than once. The purpose of calculating SESs for both top overall performances and top performances by athlete was twofold. First, this removed athlete outliers, who recorded multiple top-50 world records. Second, this allowed for more data to be included in the SES analysis. These data are reported in Table 1 as the mean SES for world-record performances overall, world-record performances by athlete, and as an aggregate of both categories.

**TABLE 1:** Sex equity score (SES) data by individual event.

**Event**	**SES (performances) *N* = 6**	**SES (athletes) *N* = 6**	**SES (aggregate) *N* = 12**
Marathon	89.02 ± 0.22	88.95 ± 0.19	88.98 ± 0.19
Half-marathon	89.22 ± 0.54	88.89 ± 0.60	89.06 ± 0.57
10 km	88.37 ± 0.21	88.36 ± 0.38	88.37 ± 0.29
5 km	88.90 ± 0.17	88.55 ± 0.25	88.72 ± 0.27
3 km	88.50 ± 0.21	88.20 ± 0.29	88.35 ± 0.28
1500 m	88.49 ± 0.25	88.81 ± 0.32	88.64 ± 0.32
800 m	88.63 ± 0.06	88.69 ± 0.19	88.66 ± 0.14
400 m	89.64 ± 0.19	89.42 ± 0.17	89.53 ± 0.20
200 m	90.19 ± 0.28	90.20 ± 0.26	90.19 ± 0.26
100 m	90.99 ± 0.06	91.26 ± 0.10	91.13 ± 0.17
60 m	92.18 ± 0.09	92.01 ± 0.10	92.10 ± 0.13
HJ	85.51 ± 0.06	85.21 ± 0.32	85.36 ± 0.27
LJ	84.84 ± 0.12	84.01 ± 0.28	84.42 ± 0.48
TJ	84.69 ± 0.04	84.34 ± 0.50	84.52 ± 0.38

*The SES is a ratio of the gap that exists between male and female performances at any given athletic event; an SES of 100.00 would indicate a sport with no gap between men and women. SES (performances) indicates data taken from the top 50 all-time performances, SES (athletes) indicates data taken from the top 50 all-time performances without multiple records per athlete, and SES (aggregate) is an aggregate of both sets of data. Abbreviated events are as follows:10-kilometer run (10 km), 5-kilometer run (5 km), 3000-meter race (3 km), 1500-meter race (1500 m), 800-meter dash (800 m), 400-meter dash (400 m), 200-meter dash (200 m), 100-meter dash (100 m), 60-meter dash (60 m), high jump (HJ), long jump (LJ), and triple jump (TJ).*

### Sex Equity Score by Event Category

After determining the 12 SES’s for each event, the 14 events were sorted into four event categories (EC’s): long-distance running, medium-distance running, short-distance running, and jumping. The three long-distance running events were the marathon, the half-marathon, and the 10-kilometer run (10 km). The four medium-distance events were the 5-kilometer run (5 km), the 3000-meter race (3000 m), the 1500-meter race (1500 m), and the 800-meter race (800 m). The four short-distance running events were the 400-meter dash (400 m), the 200-meter dash (200 m), the 100-meter dash (100 m), and the 60-meter dash (60 m). The three jumping events were the high jump (HJ), long jump (LJ), and triple jump (TJ). All records were taken for outdoor events except for the 60 m, which is an exclusively indoor event, and the 3000 m.

This sorting led to 36 SES data points for the long-distance running EC, 48 data points for the medium-distance running EC, 48 data points for the short-distance running EC, and 36 data points for the jumping EC. To determine significance across EC’s, the aggregate SES for each EC (*N* = 36 for long-distance running and jumping; *N* = 48 for medium- and short-distance running) was compared to the aggregate SES of every other EC to determine whether there were significant SES differences between EC’s. Our purpose was to test whether or not the mean changes depending on the EC. We then ran ANOVA between EC’s, as it is summarized in Table 2, where we report the corresponding *p*-values. Significance was set at *P* < 0.01.

**TABLE 2:** *P*-values of sex equity score differences between track and field event categories (EC’s).

**Event categories being compared**	**Aggregate SES (EC 1)**	**Aggregate SES (EC 2)**	***P*-value**
Long distance/medium distance	LD: 88.80 ± 0.49	MD: 88.59 ± 0.29	0.018
Long distance/short distance	LD: 88.80 ± 0.49	SD: 90.74 ± 1.00	<0.00001
Medium distance/short distance	MD: 88.59 ± 0.29	SD: 90.74 ± 1.00	<0.00001
Long distance/jumping	LD: 88.80 ± 0.49	J: 84.77 ± 0.57	<0.00001
Medium distance/jumping	MD: 88.59 ± 0.29	J: 84.77 ± 0.57	<0.00001
Short distance/jumping	SD: 90.74 ± 1.00	J: 84.77 ± 0.57	<0.00001

*Event categories are groupings of events classified as either long-distance running, medium-distance running, short-distance running, or jumping. The first EC is listed in Column 2, “Aggregate SES (EC 1).” The second EC is listed in Column 3, “Aggregate SES (EC 2).” Results in bold indicate *P*-values < 0.01.*

### Participation Disparity and Sex Equity Score

Data provided by the 2018–2019 High School Athletics Participation Survey were used to determine the degree to which the Participation Disparity (PD) between male and female athletes across different sports might act as a confounding factor and affect the SES (The National Federation of State High School Associations, 2019). The PD was calculated as a ratio of the total number of female high school athletes to the total number of male high school athletes in the same sport. This ratio was subtracted from 1 and the result multiplied by 100 to better illustrate the PD, so that a higher PD was correlated with a greater disparity between the number of male and female participants. The PD was calculated twice in total, once for cross-country high school athletes and once for outdoor track and field high school athletes.

$$\text{PD=}{\left[1-(\text{total}\text{high}\text{school}\text{female}\text{athletes}/\text{total}\text{high}\text{school}\text{male}\text{athletes)}\right]}^{*}100$$

The SES was also computed for two high school running events, the 2018 high school cross-country (HS XC) 5 km championships and the 2019 high school outdoor track (HS OT) 100-meter dash championships, using the earlier SES formula for chronometric events. The data regarding the and were collected online from the Foot Locker Cross-Country Championships and the New Balance Outdoor Nationals, respectively (Foot Locker Cross-Country Championships, 2018; New Balance Nationals Outdoor, 2019). These data were of lower quality than the data offered by the IAAF. Only 40 timed results in total were available from the 2018 boys’ HS XC championships, and 39 timed results were available from the 2018 girls’ HS XC championships. Only 41 timed results were available from the 2018 boys’ HS OT 100-meter dash preliminary finals, and 31 timed results were available from the 2018 girls’ HS OT 100-meter dash preliminary finals. The 100-meter dash preliminary finals results were used because only the top eight athletes from the preliminaries were permitted to compete in the finals; collecting data from the finals would have limited the quality of the data even further.

Because the data were limited, the SES was calculated only four times for each high school event, the first event being the HS XC 5 km and the second being the HS OT 100 m. The boys’ and girls’ results were first equalized so that the total number of male results were equal to the total number of female results in each event. This left 39 results for each sex for the HS XC 5 km and 31 results for each sex for the HS OT 100 m. The first SES calculation for each event compared the MTS_m_ to the MTS_w_ for all of the results listed in each sport. Then the results were split into terciles, so that the MTS_m_ of the top tercile of male scores was compared to the MTS_w_ of the top tercile of female scores; this was repeated for all three terciles. This process yielded four total SES calculations for each of the two high school events.

## Results

### By Individual Event

**FIGURE 1:**
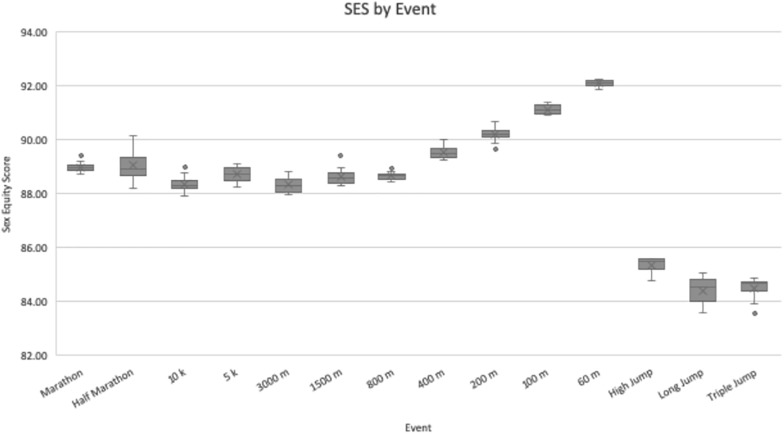
Sex equity scores by event. For each event, *N* = 12 data points were used to calculate values. Values are presented as the median (central horizontal line), mean (X), first and third quartiles (box), and minimum/maximum values (whiskers), with outliers as individual points outside of the whiskers.

Results of the SES for each event are displayed in Table 1, and all 12 SES data points are illustrated as a boxplot in Figure 1. Because the SES measures how close women are to men at a given athletic event, it is inversely correlated with the magnitude of that event’s PG so that a higher SES aligns with a more equitable sport. The aggregate SES by event ranged from a low of 84.42 ± 0.48 for the LJ to a high of 92.10 ± 0.13 for the 60-meter dash. The three lowest aggregate SES’s all belonged to jumping events, whereas the four highest SES’s all belonged to short-distance running events.

### Between Event Categories

**FIGURE 2:**
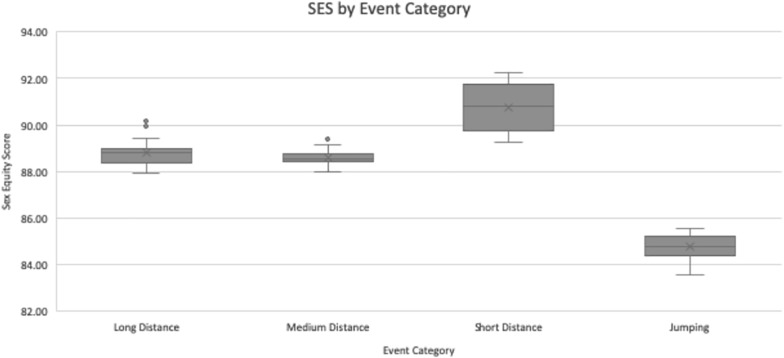
Sex equity scores by event category. For long distance and jumping *N* = 36, for medium distance and short distance *N* = 48. Values are presented as the median (central horizontal line), mean (X), first and third quartiles (box), and minimum/maximum values (whiskers), with outliers as individual points outside of the whiskers.

The real SES data for all EC’s is displayed in Table 2. The aggregate SES by EC was lowest for jumping, at 84.77 ± 0.57. Medium-distance running was next, at 88.59 ± 0.29. Long-distance running followed, at 88.80 ± 0.49. Finally, the highest SES was 90.74 ± 1.00 for short-distance running. This is illustrated in Figure 2. On the basis of this data, one can strongly conclude the average SES is significantly different for running events versus jumping events, at *P* < 0.01. Within the running events, there seem to be discrepancies: when pairwise compared, one can only fail to reject the null hypothesis that the means are uniform in the case of long versus medium distance. While there was no statistical difference in SES between long- and medium- distance EC’s, the difference between each of those events and the short-distance EC was statistically significant, at *P* < 0.01. This suggests that short-distance events exhibit a smaller PG between the sexes as opposed to events of long and medium distances.

### Participation Disparity

**FIGURE 3:**
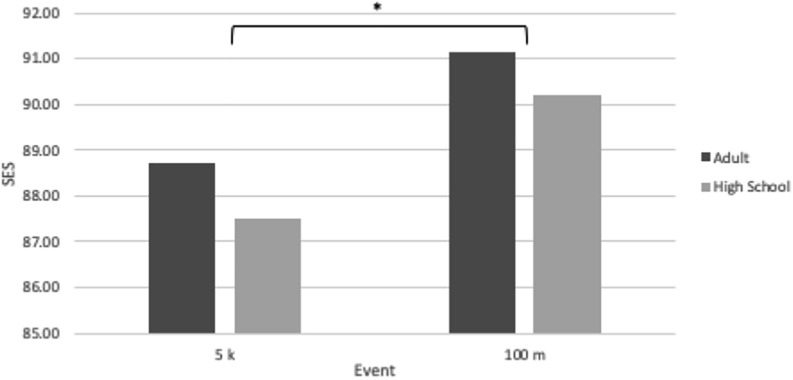
Sex equity scores for adult and high school athletes in two events: the 5 k and the 100 m. The symbol “^∗^” indicates significantly different values between events when comparing events within the same population pool, “Adult” or “High School” (*P* < 0.01).

Participation disparity data are shown in Table 3. The PD was 18.55 for HS XC and 19.34 for HS OT. The PD is useful in the context of the SES as a way to try and control for a confounding factor, so the SES for each of these high school sports is also displayed in Table 3. To better illustrate SES differences between these short- and medium-distance events, both for high school and adult athletes, the high school SES’s and the corresponding IAAF SES’s for the 5 km and the 100 m are displayed in Figure 3.

**TABLE 3:** Participation disparity (PD) in high school cross-country versus high school outdoor track and field.

	**Cross-country**	**Outdoor track and field**	***P*-value**
Male athletes	269,295	605,354	–
Female athletes	219,345	488,267	–
Ratio (F/M athletes)	0.8145	0.8066	–
PD (1-Ratio)^∗^100	18.55	19.34	–
SES	87.49 ± 1.06	90.19 ± 0.21	0.0025

*The PD measures the ratio of male to female participants at any given sport, with a PD of 0.00 indicating an equal number of male and female athletes. The SES measures the performance gap between male and female athletes at each high school sport.*

## Discussion

While some studies have explored the PG in different sports, (Thibault et al., 2010; Millard-Stafford et al., 2018; Sandbakk et al., 2018) this is the first to compare the top 50 world records in different track and field events between men and women to measure the gap that exists in these events. There was a clear difference in male versus female performance in all of these events, but the magnitude of this athletic gap ranged from 84.42 ± 0.48 for the LJ up to 92.10 ± 0.13 for the 60-meter dash. Short-distance running had a smaller PG, measured as a greater SES, than either of the other running distances, whereas jumping had a larger gap in performance than any type of running.

This study supports the idea that the PG might be used to measure the evolutionary necessity of different movements. This would be a key finding, as anthropologists and evolutionary biologists often debate the movements to which the human body and earlier hominids adapted. Our reasoning is that adaptations which increase the quality of movement of an organism, and thus the organism’s reproductive fitness, are especially selected for regardless of the organism’s sex. As a result, SBMA’s are more likely to increase the quality of movement in an organism’s offspring than are sex-dependent adaptations. Over time, these SBMA’s accumulate to advantage evolutionarily critical movements, resulting in a smaller gap in athletic ability between males and females at these and similar movements. Whether it is due to a greater degree of neuromuscular control, more efficient muscular recruiting patterns, or some combination of other physiological factors, it appears that the human body is better equipped across sex lines for a movement like short-distance running versus a movement like jumping.

The significant differences in SES across track and field EC’s suggest that humans have accrued SBMA’s which favor short-distance running over medium- and long-distance running, and every sort of running over jumping. Early humans needed to be able to quickly escape from rapidly approaching predators, similar to how modern apes in the wild are noted to have been killed by larger animals (Boesch, 1991; Tsukahara, 1993), a selective pressure that favored individuals who were good sprinters. This also could explain the SES trend within short-distance events, which was found to be inversely correlated with distance. The highest SES was 92.10 ± 0.13 for the 60 m, and the SES decreased as distance increased, down to 91.13 ± 0.17 for the 100 m, 90.19 ± 0.26 for the 200 m, and 89.53 ± 0.20 for the 400 m. The downward trend suggests that when escaping a predator, an early human’s ability to accelerate quickly, within as little as 60 m, may have determined its survival.

Long- and medium-distance running each had a smaller SES than short-distance running (88.80 ± 0.49 and 88.59 ± 0.29, respectively, compared to 90.74 ± 1.00), which would suggest that early humans’ survival and overall fitness depended more on sprinting than on longer running. The ability to run long distances was a useful trait (Bramble and Lieberman, 2004), as it allowed early bipedal hominids to travel great distances in pursuit of food or more favorable living conditions (Liebenberg, 2006). Still, an early human’s ability to sprint would have determined its survival in an encounter with a larger predator. As such, the ability to run long distances at a greater speed was a less selected-for trait, and as a result the modern human musculature is less adapted to running longer distances than it is to a single quick sprint.

Jumping, on the other hand, was considerably less useful to terrestrial hominids than any kind of running, which is reflected in the low SES’s for all jumping events. The aggregate SES for the jumping EC was significantly lower than that of any running EC, *P* < 0.01, which suggests that humans have accrued a greater number of SBMA’s in service of running rather than jumping. The contrast between short-distance running and jumping in our results is of particular note. Short-distance and jumping events alike involve quick bursts of speed and anaerobic energy expenditure (Kasabalis et al., 2005; Saraslanidis et al., 2009); during the LJ, for instance, athletes tend to sprint approximately 40 m to increase their speed before leaping (Long Jump, 2016). The major difference between an event like the LJ and the 60-meter dash, then, is the jump at the end, and this variable is enough to cause a significant drop in the SES between the two events. This suggests that the duration of a movement does not correlate with how equitable that movement is between the sexes, otherwise the SES of jumping events would be greater, perhaps even in line with the SES of short-distance running, to reflect the brief duration of a jumping event. Instead, it is another factor – the presence of SBMA’s – that shrinks the PG.

The SES has the potential to complement existing analytical tools, like surface electromyography, to determine movements to which the human body is adapted. Surface electromyography has been used to compare activity levels of muscles across different movements, which has led some researchers to conclude that jumping, which has a higher median amplitude of lower-body muscle contraction than sprinting or endurance running, reflects a type of movement to which *H. sapiens* may have adapted (Carrier et al., 2015). The results of our study, however, contradict that conclusion. Although jumping may have a greater median muscle activation than running, it is running that exhibits a smaller PG, suggesting that a greater number of SBMA’s have accumulated in the human body in service of running rather than jumping. Additionally, running can be sustained for a longer duration than jumping, which could explain how jumping, though it shows a greater relative median peak muscle amplitude than running, involves less overall muscle recruitment over an extended period of time. Far from merely contradicting the use of surface electromyography, the PG can be used alongside it to determine the evolution of the genus *Homo*. Gluteus maximus activity, for instance, is higher in endurance running than it is in walking (Lieberman et al., 2006), and the muscle is even more active in sprinting than it is in endurance running (Bartlett et al., 2013). A large gluteus maximus that developed to facilitate sprinting is therefore an example of a SBMA, which could explain the significantly smaller PG in short-distance events versus long-distance events and the higher levels of gluteus maximus engagement during sprinting versus endurance running or walking. This shows how the SES could serve as a tool alongside existing analyses, such as measurements of muscle activation, to better understand the evolution of the human musculoskeletal system.

Some confounding variables regarding the SES should be addressed here. The first is the potential for a difference in PD across events to affect our results. The greater the PD in overall male to female participation, the more likely it is that the PG increases for that event. Hypothetically, if 1,000 men competed in fictional Event A while only 500 women did the same, versus if 1,000 men and 1,000 women competed in fictional Event B, we would expect Event A to have a lower SES than Event B, because the top 50 female scores would be drawn from half the population size in Event A versus Event B. If women are more inclined to participate in certain sports (like running) rather than others (like jumping), the SES would be inflated in the events with more female participants. Research documenting a general disparity between general male and female participation rates in athletics does exist (Deaner et al., 2012; Drake et al., 2015), but the literature does not specifically suggest that a participation gap exists between different track and field events.

To control for this, we examined the PD as it related to two running events: the 5 km and the 100 m. These two events were compared because they fell into different EC’s – medium-distance running for the 5 km and short-distance running for the 100 m – and thus would be expected to possess significantly different SES’s. Because there was no available IAAF data on participation rates amongst world-record athletes, we used high school athletics statistics to measure the PD between male and female athletes in two sports: HS XC and HS OT. The PD for HS XC was calculated as 18.55, whereas the PD for HS OT was calculated as 19.34, meaning there was a greater PD for HS OT events like the 100 m versus for HS XC events like the 5 km. Figure 3 illustrates that, while high school running events appear to have smaller SES’s than adult events, the SES difference between the adult 100 m and the adult 5 km (2.41 ± 0.44) is relatively similar to the SES difference between the high school 100 m and the high school 5 km (2.70 ± 1.27).

If a greater proportion of males compete in any given event, the PG between the male performance and female performance should widen, too. This does not appear to be the case for high school running events. The PD is greater for HS OT versus HS XC, yet the 100 m, a HS OT event, has a greater SES – indicating a smaller PG – than the 5 km, a HS XC event. Though HS OT has a greater PD than HS XC, its PG is smaller between men and women, as evidenced by the SES of 90.19 ± 0.21 for the high school 100 m versus the SES of 87.49 ± 1.06 for the high school 5 km. The PG is smaller in this short-distance running event even though the PD is greater, which indicates that the proportion of male to female participants is not likely to be as impactful as the type of track and field event on the magnitude of the PG. While these data provide substantial evidence that the PD is greater in HS OT than HS XC, the data from the 2018–2019 High School Athletics Participation Survey lumps all HS OT participants together, rather than just athletes who participate in the 100 m, so there is still room for error in the calculation of this PD.

However, the differences in event performances by sex between events appear to remain stable when looking at track and field competition across all levels. At the 2018 National Collegiate Athletic Association (NCAA) Division I track and field championships, for instance, the PG between the top-scoring woman and the top-scoring man followed the same general pattern as that of all-time track and field records calculated in this paper. It should be noted that in the case of the NCAA championships, only the top results are listed for men and women, which leaves more room for error when computing the magnitude of the PG for its events. The NCAA PG is 92.01 for the 100-meter dash (vs. 91.13 ± 0.17 all time), 88.87 for the 10-kilometer run (vs. 88.37 ± 0.29), and 83.62 for the TJ (vs. 84.52 ± 0.38) (NCAA Division I Championships, 2018). More research should be done on equally sized samples of male and female populations, including, for instance, untrained individuals to confirm that the magnitude of the PG between men and women remains stable for these track and field events.

The main issue with the PG as a tool to measure evolutionary movement is that in some cases, the magnitude of the gap appears to be notably unrelated to the evolutionary history of *H. sapiens*. Swimming, for instance, has a PG similar to – and in some cases smaller than – some running events (Millard-Stafford et al., 2018), even though swimming lacks the evolutionary record that running does in humans (Bramble and Lieberman, 2004; Castillo and Lieberman, 2018). This is in part due to the difference in fat distribution between men and women; females hold more subcutaneous fat in their lower body, which aids in buoyancy and therefore improves their swimming capacity (Blaak, 2001; Karastergiou et al., 2012). This differing subcutaneous fat distribution reduces the magnitude of the PG for swimming, even though running has been more evidently useful for humans on an evolutionary level. However, the world records kept for swimming are sparser than those kept by the IAAF for track and field, so the swimming data may be particularly affected by outliers. Still, the SES alone should not be used to measure how evolutionary a movement might be; the PG needs to be examined alongside other components of the evolutionary record to determine whether a smaller disparity between the sexes is truly due to SBMA’s or whether the gap is caused by a factor unrelated to evolution.

## Conclusion

The SES is a new approach to the evaluation of movement that could help clarify the movements essential to the evolution of the human musculoskeletal system. By applying it to 14 track and field events, we see that running has a significantly smaller disparity between the sexes than jumping does. Furthermore, within running events, short-distance running exhibits a significantly smaller PG than do medium- or long-distance running. The magnitude of this PG could be explained by the importance of SBMA’s in early humans. Because SBMA’s are more efficient at improving the quality of movement across generations than sex-specific adaptations, the result is a smaller gap between male and female performance in the track and field events most similar to movements early humans engaged in as a result of some selective pressure. While more research on the PG in sport is needed, it has the potential to evaluate movements to which the human musculoskeletal system adapted, which in turn can help clarify the evolutionary history of *H. sapiens* and inform exercise scientists as to which movements the human body is best-suited.

## Data Availability Statement

All datasets generated for this study are included in the article/Supplementary Material.

## Author Contributions

CC is the sole author of this manuscript, and the hypothesis described here is entirely his own.

## Conflict of Interest

The authors declare that the research was conducted in the absence of any commercial or financial relationships that could be construed as a potential conflict of interest.
